# Larvicidal effects of Chinaberry (*Melia azederach*) powder on *Anopheles arabiensis *in Ethiopia

**DOI:** 10.1186/1756-3305-4-72

**Published:** 2011-05-10

**Authors:** Ryan E Trudel, Arne Bomblies

**Affiliations:** 1 University of Vermont Department of Civil and Environmental Engineering 33 Colchester Ave. Burlington, VT 05405 USA

## Abstract

**Background:**

Synthetic insecticides are employed in the widely-used currently favored malaria control techniques involving indoor residual spraying and treated bednets. These methods have repeatedly proven to be highly effective at reducing malaria incidence and prevalence. However, rapidly emerging mosquito resistance to the chemicals and logistical problems in transporting supplies to remote locations threaten the long-term sustainability of these techniques. Chinaberry (*Melia azederach*) extracts have been shown to be effective growth-inhibiting larvicides against several insects. Because several active chemicals in the trees' seeds have insecticidal properties, the emergence of resistance is unlikely. Here, we investigate the feasibility of Chinaberry as a locally available, low-cost sustainable insecticide that can aid in controlling malaria. Chinaberry fruits were collected from Asendabo, Ethiopia. The seeds were removed from the fruits, dried and crushed into a powder. From developmental habitats in the same village, *Anopheles arabiensis *larvae were collected and placed into laboratory containers. Chinaberry seed powder was added to the larval containers at three treatment levels: 5 g m^-2^, 10 g m^-2 ^and 20 g m^-2^, with 100 individual larvae in each treatment level and a control. The containers were monitored daily and larvae, pupae and adult mosquitoes were counted. This experimental procedure was replicated three times.

**Results:**

Chinaberry seed powder caused an inhibition of emergence of 93% at the 5 g m^-2 ^treatment level, and 100% inhibition of emergence at the two higher treatment levels. The Chinaberry had a highly statistically significant larvicidal effect at all treatment levels (χ^2 ^= 184, 184, and 155 for 5 g m^-2^, 10 g m^-2 ^and 20 g m^-2^, respectively; p < 0.0001 in all cases). In addition, estimates suggest that sufficient Chinaberry seed exists in Asendabo to treat developmental habitat for the duration of the rainy season and support a field trial.

**Conclusions:**

Chinaberry seed is a very potent growth-inhibiting larvicide against the major African malaria vector *An. arabiensis*. The seed could provide a sustainable additional malaria vector control tool that can be used where the tree is abundant and where *An. arabiensis *is a dominant vector. Based on these results, a future village-scale field trial using the technique is warranted.

## Background

Malaria continues to claim lives in African villages, despite repeated control programs that have reduced, but not eliminated, morbidity and mortality from the disease. The typical national- or international-level control programs targeting adult mosquitoes in villages depend on synthetic insecticides for indoor residual spraying (IRS) or insecticide treated bednets (ITN). Repeated successes in reducing malaria burden using these methods to target adult mosquito longevity have contributed to the near abandonment of historically-favored techniques seeking to eliminate sub-adult mosquitoes in their aquatic developmental habitats, and have justifiably reinforced the role of ITN and IRS as the preferred primary malaria intervention methods. However, emerging mosquito resistance to permethrin and deltamethrin used in ITNs and the dichlorodiphenyltrichloroethane (DDT) used in IRS [1-5] threatens the long-term sustainability of IRS- and ITN-based interventions. Increased resistance to the insecticide's knock-down effect will likely reduce the long term efficacy of these methods in suppressing malaria transmission. Moreover, ITNs and IRS-based malaria control techniques require complicated logistics for distribution of the products, and may not always easily reach the rural, impoverished people that they are intended to protect [6]. It may therefore be beneficial to the rural communities to supplement external malaria control programs with a method that is independent of outside support, is not susceptible to development of resistance which threatens long-term sustainability, and is low-cost yet effective in reducing vector abundance. It is hoped that Chinaberry can provide such a solution.

Resistance to insecticides is a serious problem threatening malaria control efforts in all regions where insecticides are used to kill mosquitoes. Near Jimma, Ethiopia, Yewhalaw *et al. *[5] noted a 99% resistance among adult *An. arabiensis *mosquitoes to DDT, 82% resistance to permethrin and 18% resistance to deltamethrin. This emergent resistance renders local IRS programs using DDT virtually ineffective, and the efficacy of permethrin and deltamethrin diminished. As a defense against the development of such resistance, some plant species evolve to contain more than one insecticidal chemical, in order to maintain protective effects against invertebrates. For example, the neem tree (*Azadiracta indica*) contains 99 active chemicals that are known to have insecticidal properties [7], a characteristic that makes the emergence of resistance extremely unlikely. Azadiractin is the most active of these chemicals, and is difficult to synthesize and very complex in nature [8]. *Azadiracta indica *(neem) and *Melia azederach *(Chinaberry) have many similar properties and are very similar morphologically [8,9]. The genera *Azadirachta *and *Melia *are sister taxa within the *Meliaceae *family and are thus closely related in an evolutionary sense [10]. This close relationship suggests that the Azadiractin-producing Chinaberry tree has evolved a similar combination of active ingredients to ensure sustainable insecticidal activity to protect itself from insects. While this has not been proven, evidence that the tree continues to have insecticidal effects and that insects have not developed resistance suggests that this is the case. Chinaberry extract has been shown to be an effective growth-inhibiting larvicide when tested against *Aedes aegypti *and *An. stephensi *larvae as well as several other pests [9,11-13]. Azadiractin (as well as other extracts such as meliacarpinin) does not have a knock-down effect; rather it is a true growth inhibiting larvicide because it interferes with the hormonal control of metamorphosis [11].

Chinaberry is found in parts of Asia and Africa in a range of climatic conditions. The tree's range often overlaps with malaria-endemic zones, and the tree is also abundant in the Ethiopian highlands. Chinaberry is often planted in Africa because it is fast growing, a good source of firewood and people welcome the shade it provides in arid conditions [14].

Previous field trials have shown the potential of village-sourced insecticidal plants to help control malaria in a small community, independent of outside aid. For example, Okumu *et al. *[15] tested oils from the neem tree (*Azadiracta indica*) against third instar larvae of the major African malaria vector *An gambiae ss*. They noted high levels of emergence inhibition at low neem oil concentrations. In a similar study, Gianotti *et al *[16] showed that the aqueous extract of the neem tree's seeds could suppress adult mosquito abundance in a village in Niger. The neem seeds in that study were crushed and sprinkled on the surface of developmental habitats at 10 g m^-2^. Mosquito larvae ingested the crushed seed, thus becoming affected by the growth-inhibiting property of the active chemicals in the seed. The authors effectively demonstrated a reduced abundance of *An. gambiae *adults in the village where neem seed was applied to aquatic developmental habitat, compared with a control village where anopheline abundance was monitored but neem was not applied. The study did not evaluate the efficacy of crushed neem seed in a carefully controlled laboratory environment, and the inhibition of emergence is not exactly known. However, because neem and Chinaberry are closely related, the successful method of field application described in Gianotti *et al. *[16] may be effective when using Chinaberry seeds where that tree is abundant and where neem is absent.

In this study we confirm and quantify the potency of Chinaberry in preparation for such a study. We present laboratory evidence that the powdered seed of the Chinaberry tree (*Melia azederach*) can decrease mosquito numbers through a strong growth-inhibiting larvicidal effect when applied to aquatic developmental habitat of the common African malaria vector *Anopheles arabiensis*. The result is a method of malaria mosquito population control that does not depend on outside support, and may provide villagers with a sustainable, ecologically sound means of reducing mosquito abundance. The abundant Chinaberry tree may therefore offer a potential sustainable additional malaria control tool that does not suffer from the aforementioned problems with ITN and IRS.

## Methods

### Study Site

The study was conducted in the village of Asendabo, Ethiopia (7° 45' N, 37° 13' E, elevation 1750 meters). Asendabo has approximately 1000 inhabitants, many of whom are subsistence farmers. The center of the village is made up of densely clustered houses, and Chinaberry trees are commonly found in these residential areas. The Ethiopia Ministry of Health employs an IRS program for malaria vector control, and distributes ITNs [5,17]. We observed ITN usage to be moderate in the village, but several houses were observed to have visible DDT on the walls. Asendabo is located in the highlands approximately 50 km from Jimma, and receives about 700mm of rain per year, much of it falling during the June - September rainy season. During the wet season, the average temperature is approximately 20 degrees. Subsistence dryland agriculture dominates food production, and local vegetation consists primarily of teff, pepper and maize crops. Most of the land is farmed, some is left fallow, and the remainder consists of pastureland with stands of eucalypts and other trees. Malaria transmission at the site is endemic but seasonal, and mosquito populations increase dramatically as a result of the monsoon rains. *An. arabiensis *is the dominant malaria vector, and breeds prolifically in the borrow pits commonly found near houses. These borrow pits are created when people 'borrow' clayey mud from near their homes and shore up walls of their homes. Because they are almost always very close to human habitation, mosquito development in these pools constitutes a very dangerous situation. The sub-adult mosquitoes used in the lab tests were collected from such borrow pits. During the rainy season, these borrow pits typically contain several inches of turbid water in which *An. arabiensis, An. coustani*, and *Culex spp *mosquito larvae are all found. These borrow pit pools persist from mid- July through the end of the rainy season in September or early October. These pools do not contain complex ecosystems and the water is not used as a domestic water supply.

**Figure 1:**
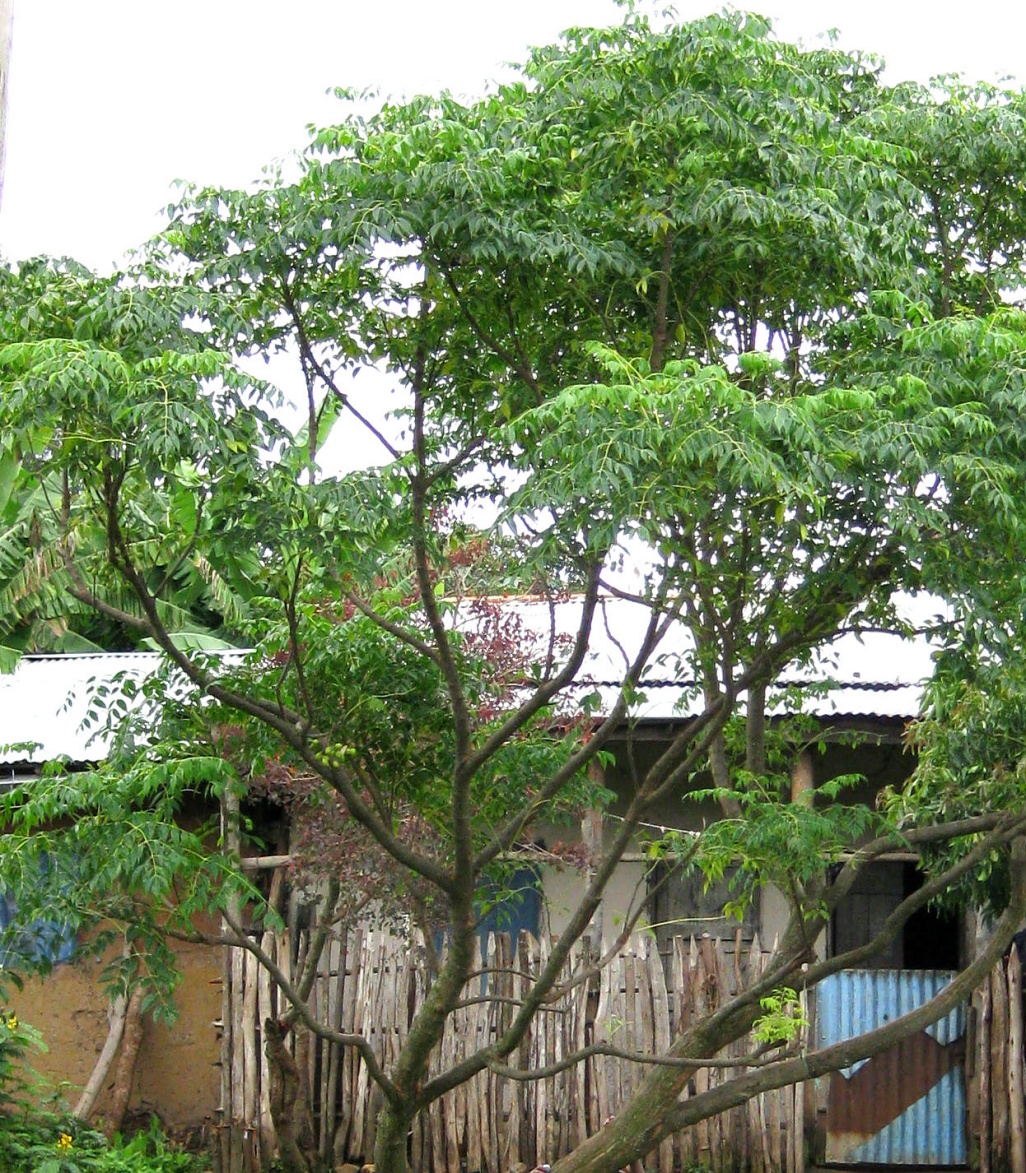
**A Chinaberry (*Melia azederach*) tree in Asendabo**. A small amount of fruit was found in this particular tree, but fruiting yields were seen to vary greatly among trees.

The Chinaberry seeds collected for this experiment grew in abundance throughout Asendabo. A survey of the village was conducted in order to gauge the estimated yearly fruit yield of all the Chinaberry trees in the village. 434 Chinaberry trees were counted in Asendabo, a village with an area of 2 × 10^6 ^m^2^. This was observed to be a typical tree density in the Jimma region. The trees were observed to bear fruit during the rainy season, with abundant ripe fruit during July 2010, when the study was carried out. The local residents generally ignore the fruit, letting it fall to the ground, unused. The seeds are left to decompose in the environment. Dried seeds are also occasionally used to make jewelry. Villagers burn the tree's leaves to repel mosquitoes, and they brew a tea from the leaves to treat the symptoms of malaria. An example of a medium-sized Chinaberry tree in Asendabo is shown in Figure 1.

### Seed preparation

**Figure 2:**
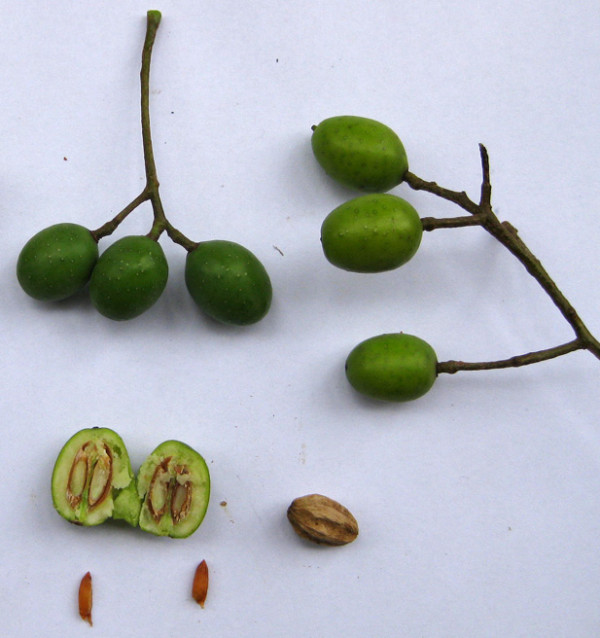
**The berries and seeds of the Chinaberry tree, *Melia azederach***.

Chinaberry fruit was gathered with the help of locals from trees growing in village common areas. This involved simply gathering several kilograms of fallen fruits below the trees, and occasionally picking low-hanging fruit from the trees. The collected fruit was dried in the sun and the soft outer flesh was removed by hand by squeezing each fruit. The de-pulped seed casings were then dried for seven days in the shade before being crushed and powdered using a wooden mortar and pestle. This tool was purchased at a market in Jimma in order to avoid using a mortar and pestle that would otherwise have been used for food production in Asendabo. Care was taken to complete the seed preparation using only local tools, in order to demonstrate that outside supplies and tools of any type are not necessary to implement the described approach. The dried, powdered seeds were stored in a dry, shaded plastic container for several days prior to use in the laboratory trials. Chinaberry fruits, seeds, and seed pods are shown in Figure 2. Three separate batches of Chinaberry powder were prepared for use in each of the separate trials.

### Laboratory trials

The laboratory trials were designed to focus directly on the relationship between larval development, eclosion and Chinaberry powder application. The trials were conducted in a lab at the local district health clinic in Asendabo. Experimental design followed the WHO's Guidelines for Laboratory and Field Testing of Mosquito Larvicides [18]. Since this study uses a granular seed powder instead of a lab derived solvent based solution, some of the procedure was adapted to allow for the use of seed powder. The seed powder effectively targets mosquito larvae in part because it floats on the water surface where the *Anopheles *larvae feed, so there was no effort to shake or stir to create a homogenous solution. Because mosquitoes feed largely on organic particulate material from the surface film of puddles, the trial was done based on weights of dried seed per unit area of water surface on which the powdered seed will float.

The *An. arabiensis *larvae collected were mostly 2^nd ^instars. From their developmental habitats (usually borrow pits), the larvae were transported directly back to the lab in plastic whirl-packs. They were placed into plastic trays for rearing and fed crushed dry bulk dog food. These sub-adult populations served as the source of third instar larvae used for the Chinaberry trials.

The trial involved a graded bioassay with 3 treatment levels of Chinaberry seed and a control group as a way of estimating the amount of Chinaberry seed needed to act as a total larvicide. The three treatment levels were 0.05g, 0.1g and 0.2g of crushed seed applied to each larval container. The larvae were transferred from the plastic trays to BioQuip™ Mosquito Breeders (purchased from BioQuip, Rancho Dominguez, California) using a dropper. For all treatment levels (control, 0.05g, 0.1g, and 0.2g), 100 larvae were divided into four batches of 25 larvae and placed into identical larval containers (BioQuip Breeders). These cylindrical containers had a surface area of 100cm^2^, with a cone shaped trap leading into a vented top compartment for containment of adult mosquitoes. The larval containers were filled with 333ml water each, with the water depth kept around 3 cm. The larvae were placed in the water, and again offered a small amount of powdered dog food. The Chinaberry powder was then sprinkled onto the water surface of the larval containers as an alternative food source. From the geometry of the containers, the three treatment levels of 0.05g, 0.1g and 0.2g correspond to 5 g m^-2^, 10 g m^-2^, and 20 g m^-2^. The temperature was ambient (average 20°C) and did not vary among the containers.

The larvae were monitored each day and the total number of 3^rd ^instars, 4^th ^instars, pupae and adults was recorded. Dead larvae were removed once they were discovered. The larval containers were thoroughly cleaned between each use and a fresh batch of larvae was used for each replicate. The experimental procedure was replicated three times, but the third replicate had to be discarded because adult emergence in the control was only 65%. The reason for the high mortality in the control is not known.

## Results

**Figure 3:**
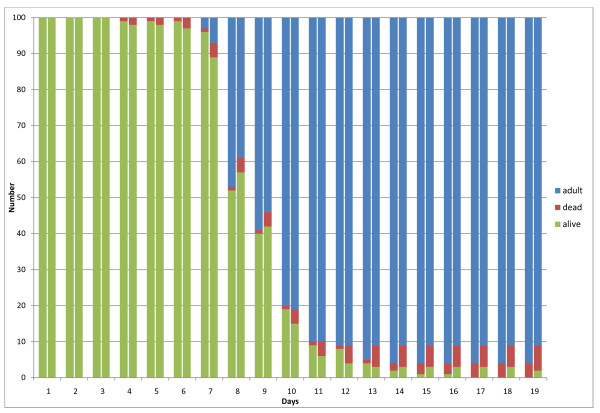
**Results from the control group**. The results show the total number of larvae or pupae that are alive (green), dead (red) or have emerged as adult mosquitoes (blue). These results are from four larval containers which contained 25 larvae each. The two columns for each day show replicates 1 (left) and 2 (right) of the experiment.

**Figure 4:**
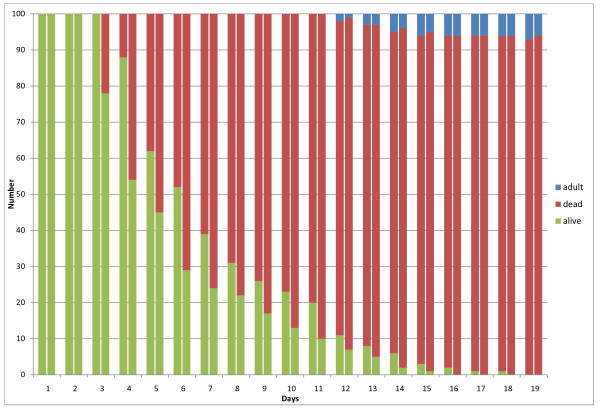
**Results from the 0.05 gram application treatment group**. The results show the total number of larvae or pupae that are alive (green), dead (red) or have emerged as adult mosquitoes (blue). These results are from four larval containers which contained 25 larvae each. The two columns for each day show replicates 1 (left) and 2 (right) of the experiment.

**Figure 5:**
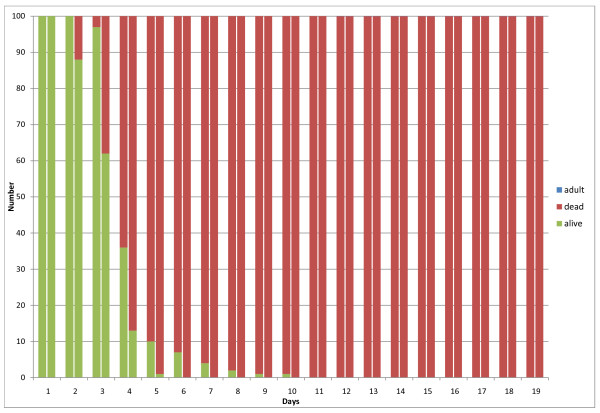
**Results from the 0.1 gram application treatment group**. The results show the total number of larvae or pupae that are alive (green), dead (red) or have emerged as adult mosquitoes (blue). These results are from four larval containers which contained 25 larvae each. The two columns for each day show replicates 1 (left) and 2 (right) of the experiment.

**Figure 6:**
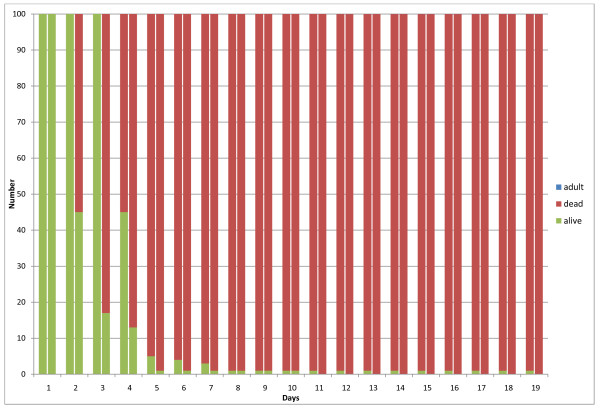
**Results from the 0.2 gram application treatment group**. The results show the total number of larvae or pupae that are alive (green), dead (red) or have emerged as adult mosquitoes (blue). These results are from four larval containers which contained 25 larvae each. The two columns for each day show replicates 1 (left) and 2 (right) of the experiment.

Trial results are presented in Figures 3, 4, 5 and 6. Figure 3 depicts the results for the control group, for the first two replicates of the experiment. For each day, the number of live larvae or pupae (grouped together), dead larvae or pupae, and adult mosquitoes is plotted. The majority of mosquito larvae emerged as adults in the control group for replicates 1 and 2, with a 4% mortality in replicate 1 and a 7% mortality in replicate 2. In addition, replicate 2 had a 2% non-emergence (live pupae that did not mature into adult mosquitoes after 19 days). For each day, all individuals (dead or alive) are accounted for to sum to the initial 100 larvae at the beginning of the experiment. Figure 4 depicts results for 0.05g ground Chinaberry seed (including seed casing), applied to 333mL of water in each larval container. Figure 5 shows similar results from the 0.1 g treatment group, and finally Figure 6 depicts the results for the 0.2 g treatment group. No adult mosquitoes emerged from the 0.1 g and 0.2 g treatment groups. Seven adult mosquitoes emerged from the 0.05g treatment group in replicate 1, and six emerged in replicate 2.

In both replicates 1 and 2, each treatment group had much higher mortality than the control group, as expected by the growth-inhibiting azadiractin activity in the Chinaberry seeds. For replicate 1, the larval mortality for each of the treatment groups was found to be at least 23 times greater than the control group. In replicate 2, larval mortality was at least 13 times greater than in the control group. The experimental results were analyzed using a chi-squared test applied to a contingency table, with one degree of freedom. Table 1 summarizes the statistics. The very high chi-squared statistics show that observed larval mortality differs greatly from expected larval mortality. In addition, these results are highly statistically significant (p < 0.0001) for all treatment levels in both replicates, indicating that there is less than 0.001% chance that the observed results could have arisen if Chinaberry seed had no effect. The inhibition of emergence (IE) is a measure of efficacy of the applied growth-inhibiting larvicide in preventing adult mosquito emergence from the developmental habitat. IE is given by the formula:

**Table 1:** Summary of the chi-squared statistic, associated p-value, and inhibition of emergence for each treatment level, for both experimental replicates

	**REPLICATE 1**			**REPLICATE 2**		
	
	**0.05 g**	**0.1 g**	**0.2 g**	**0.05 g**	**0.1 g**	**0.2 g**
	
χ^2^	184	184	155	184	184	155
p	<0.0001	<0.0001	<0.0001	<0.0001	<0.0001	<0.0001
IE (%)	92.71	100.00	98.96	93.55	100.00	100.00

where T is the percentage survival in treated batches, and C is the percentage survival or emergence in the control [18]. An IE value of 100% corresponds to perfect larvicidal efficacy, and an IE of 0% would signify that the treatment had no effect. Calculated IE values from the lab trials are summarized in Table 1. The results clearly show that Chinaberry seed is highly effective at inhibiting the growth and emergence of adult *An. arabiensis *mosquitoes.

## Discussion

The presented results show that the Chinaberry tree (*Melia azederach*) has highly significant growth-inhibiting larvicidal effects on the malaria vector *An. arabiensis*, the most abundant malaria vector in Asendabo, Ethiopia. The inhibition of emergence (IE) was 100% or near 100% for all of the trials in both experimental replicates. These results are encouraging, and will support the design of a field trial in Asendabo to ascertain efficacy when this technique is applied within a village. The IE is expected to be lower in a field trial than in the carefully controlled, idealized laboratory setting. This may be partly due to wind gusts blowing the powdered seeds on the surface of larger pools to one side of the pool, leaving areas of pool surface without seed. In smaller pools this is not likely to be a significant problem. IE in the field may also be lower than in the laboratory because alternative particulate nutrient sources (such as wind-dispersed maize pollen, for example) can collect on the surface film, reducing the likelihood that anopheline larvae will take the powdered Chinaberry seed as a food source. The presence of such alternate, non-toxic nutriment sources may reduce the uptake of the powdered seed by the immature larvae. Nevertheless, the strong results of this laboratory study confirm that Chinaberry seed is a powerful growth-inhibiting larvicide when used against *Anopheles arabiensis*. The next step in this research is a field trial, conducted in Asendabo.

A ground survey of the trees in Asendabo was performed to estimate the availability of Chinaberry. There are many Chinaberry trees in the village, which ranged in trunk diameter from small sapling to approximately 50 cm in a few cases. A ground survey of Asendabo found 434 Chinaberry trees in the village of which 31% (136) held some Chinaberry fruits at the time of the survey. With approximately 15 kilograms average annual yield of fruit per mature, fruit-bearing tree, approximately 2040kg of Chinaberry fruit were available this year to Asendabo. Approximately 80% of this mass is in the fruit pulp. After removal of the pulp and drying of the seeds, 408kg of dried seedpod would remain for grinding and use as a larvicide. For the 10-week period during the peak transmission season (mid-July through September), assuming a weekly reapplication, this amount would suffice to treat 8160 m^2 ^of *An. arabiensis *developmental habitat at the 0.05 g treatment level (5 g m^-2^). The total surface area of developmental habitat in Asendabo was not measured in detail because it fluctuates, but a rough estimate can be made. Using a geographic information system, the area of Asendabo was estimated to be 2 × 10^6 ^m^2^. If the density of standing water is 1 square meter (mostly borrow pits) every 20-meter by 20-meter area (400m^2^), then 5000 m^2 ^of water surface area need treatment. This rough estimate of surface area needing treatment is less than the estimate of the pool surface area for which sufficient Chinaberry seed exists. Some of these microtopographic depressions could likely be easily filled, drained, or otherwise eliminated. The remainder could be treated with Chinaberry seeds on a weekly basis. Sufficient seed is estimated to be available for such an application rate throughout the rainy season. The tree survey of Asendabo confirms that sufficient Chinaberry seed is available to support a field trial. The methods employed in this study can be easily scaled up to target those breeding pools that are identified to host *Anopheles *mosquito larvae.

Toxicity to vertebrates is a concern, as Chinaberry has been documented to have had ill effects and even resulted in death when mammals have ingested large quantities [19-22]. However, the documented toxic quantities are many orders of magnitude greater than those used in this trial. Chinaberry trees and their fallen fruit coexist with vertebrates in Asendabo with no ill effect. Besides low concentrations of aqueous Chinaberry extracts, animals typically do not drink from the turbid, ephemeral pools that constitute the favored developmental habitat of *An. arabiensis*.

The presented results are significant primarily because they are the laboratory basis for a sustainable *Anopheles *mosquito control methodology that is independent of outside donors and can be undertaken by villagers using only locally-available supplies. While it is not expected that mosquito populations will ever completely disappear using Chinaberry seeds, dedicated programs organized from within villages could potentially significantly reduce vectorial capacity by targeting mosquito larvae in their developmental habitats. Moreover, the method promises to be free of complicated logistics, highly unlikely to stimulate resistance to Azadiractin, and thus much more sustainable than exclusive reliance on synthetic insecticides.

## Conclusions

The presented laboratory tests have shown that powdered Chinaberry (*Melia azederach*) seed has very potent growth-inhibiting larvicide properties against *Anopheles arabiensis*, the dominant malaria vector in the region of Jimma, Ethiopia as well as much of Africa. At moderate treatment levels of 5 grams per square meter of water, the inhibition of emergence was 93%, and at 10 grams per square meter, laboratory tests with 100 individuals showed complete inhibition of emergence. No adult mosquitoes emerged from treated water. A rudimentary survey suggests that enough Chinaberry seed exists in the study village of Asendabo to suppress mosquito populations and thus diminish vectorial capacity. Both of these observed results suggest that field trials using Chinaberry seed are necessary, feasible and likely to succeed. Although this method will most likely not (and probably should not) replace currently employed malaria control strategies, it may offer an additional tool to be used in an integrated approach to combat malaria that is completely sustainable. Future field studies will seek to confirm the efficacy of Chinaberry seed to combat *Anopheles arabiensis *mosquitoes at the village scale.

## Competing interests

The authors declare that they have no competing interests.

## Authors' contributions

RET conducted the field trial, participated in writing the manuscript, and participated in the data analysis. AB conceived of the study, provided supervision, participated in writing the manuscript, and participated in the data analysis. Both authors read and approved the final manuscript.
